# miRNAs in pancreatic cancer progression and metastasis

**DOI:** 10.1007/s10585-023-10256-0

**Published:** 2024-01-19

**Authors:** Ellie T. Y. Mok, Jessica L. Chitty, Thomas R. Cox

**Affiliations:** 1 Matrix & Metastasis Lab, Cancer Ecosystems Program, The Garvan Institute of Medical Research and The Kinghorn Cancer Centre, Darlinghurst, NSW Australia; 2 School of Clinical Medicine, St Vincent’s Healthcare Clinical Campus, UNSW Medicine and Health, UNSW Sydney, Sydney, NSW Australia

**Keywords:** microRNA, Pancreatic ductal adenocarcinoma, Novel therapeutics, Diagnostics

## Abstract

Small non-coding RNA or microRNA (miRNA) are critical regulators of eukaryotic cells. Dysregulation of miRNA expression and function has been linked to a variety of diseases including cancer. They play a complex role in cancers, having both tumour suppressor and promoter properties. In addition, a single miRNA can be involved in regulating several mRNAs or many miRNAs can regulate a single mRNA, therefore assessing these roles is essential to a better understanding in cancer initiation and development. Pancreatic cancer is a leading cause of cancer death worldwide, in part due to the lack of diagnostic tools and limited treatment options. The most common form of pancreatic cancer, pancreatic ductal adenocarcinoma (PDAC), is characterised by major genetic mutations that drive cancer initiation and progression. The regulation or interaction of miRNAs with these cancer driving mutations suggests a strong link between the two. Understanding this link between miRNA and PDAC progression may give rise to novel treatments or diagnostic tools. This review summarises the role of miRNAs in PDAC, the downstream signalling pathways that they play a role in, how these are being used and studied as therapeutic targets as well as prognostic/diagnostic tools to improve the clinical outcome of PDAC.

## Introduction

MicroRNA (miRNA) are small RNA molecules that play a crucial role in regulating gene expression by binding to messenger RNAs (mRNA), thereby influencing protein production and downstream cellular function. In cancer, dysregulation of miRNAs is frequently observed, contributing to disease progression, making them important biomarkers and potential therapeutic targets in cancer research and treatment. In the context of pancreatic ductal adenocarcinoma (PDAC), there is an increased in effort to study and reveal specific alterations in miRNA expression patterns and how they have contributed to the disease. Here, we shed light on the pivotal role of miRNAs in the development and progression of this difficult-to-treat cancer.

## microRNAs and their function

MicroRNAs are small non-coding RNA molecules that are known to play a crucial role in the regulation of gene expression in eukaryotic cells. They are typically between 21 and 25 nucleotides in length and have been shown to post-transcriptionally regulate gene expression by binding to mRNA molecules. This binding occurs mainly at the 3' untranslated region (3’UTR) of the target mRNA where their binding can prevent protein translation or trigger the degradation of target mRNAs.

Through controlling the expression of specific genes, miRNAs play critical roles in a wide range of biological processes, including development, cell differentiation, apoptosis and response to environmental changes and stress. Dysregulation of miRNA expression or function has been linked to various diseases, including cancer, neurodegenerative disorders, cardiovascular diseases, and metabolic disorders.

The miRNA field has significantly grown in recent years leading to mounting evidence of their biological importance. When the first miRNA, *lin-4*, was discovered in *Caenorhabditis elegans* in 1993, it was characterised as a gene responsible for regulating worm development. It was later discovered that *lin-4* was able to bind to the 3’UTR of another gene named *lin-14* and led to a post-transcriptional downregulation of *lin-14*, therefore *lin-4* was not a typical gene but a non-coding RNA responsible for the regulation of target genes [1]. Several years later, the second miRNA *let-7* was discovered and more excitingly, *let-7* was found to be conserved across species including humans [2]. As the field expanded so did the discovery of more small non-coding RNAs, now known as miRNAs. To date, there are 1,917 human miRNAs recorded in the miRNA database, miRBase [3].

**Fig. 1:**
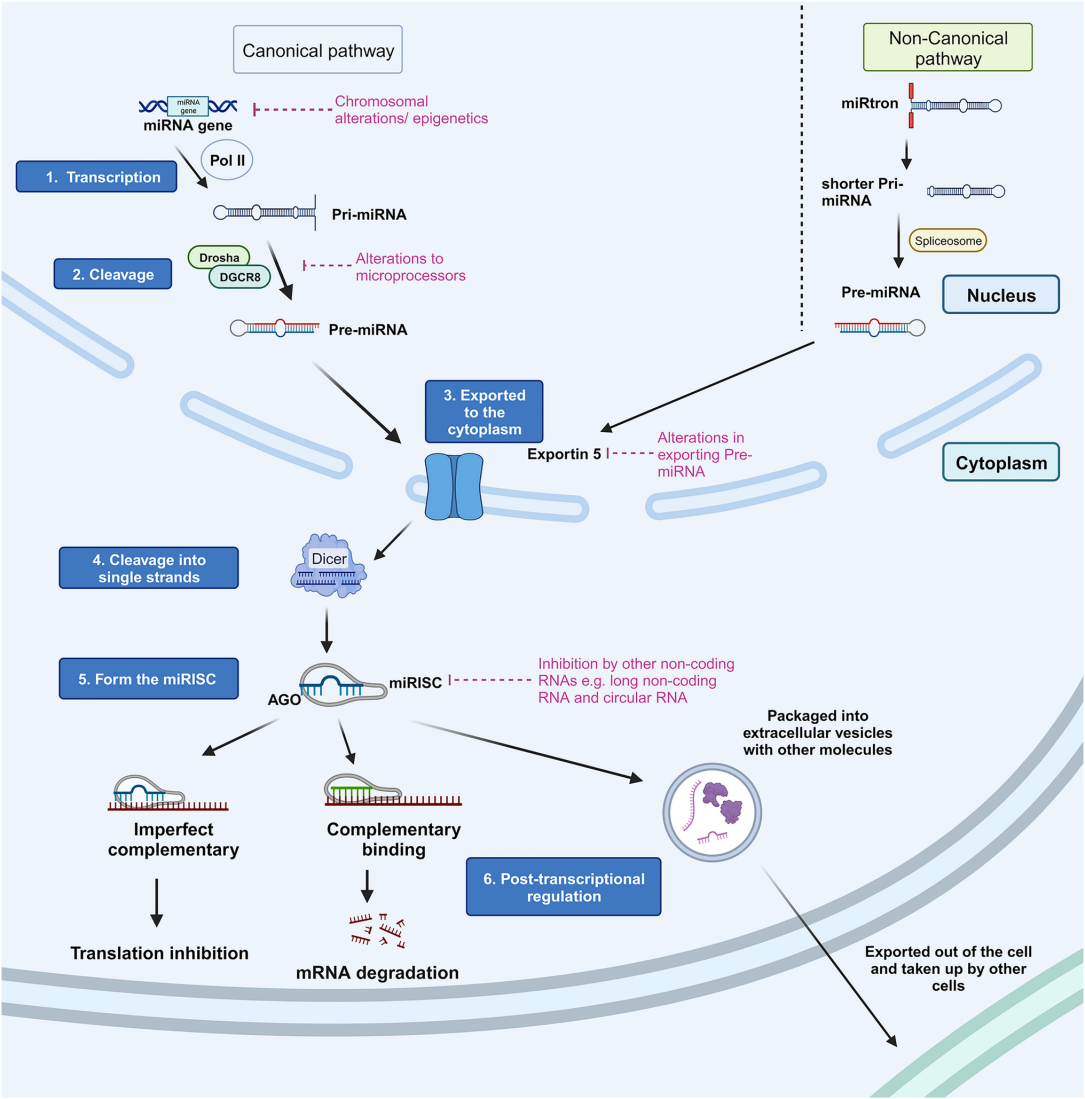
Schematic of the canonical and non-canonical pathways of microRNA (miRNA) biogenesis. Canonical pathway is carried out by 6 steps as follows: 1. miRNA transcription by polymerase II 2. Pri-miRNA cleavage by microprocessors 3. Export of pre-miRNA by Exportin 5 4. Pre-miRNA cleavage by Dicer 5. Guide strand forms a miRNA-induced silencing complex (miRISC) after attachment to AGO 6. Binding of miRISC to target mRNA or export to other cells in extracellular vesicles. Several non-canonical pathways exist, for example beginning with transcription of the miRtrons followed by splicing by debranching enzyme 1 (DBR1) resulting in pre-miRNA that then follows the export as and final cleavage per the canonical pathway steps 3–6

Since the discovery of *lin-14*, it is now well established that miRNAs can bind to the 3’UTR of mRNAs and cause mRNA degradation (complementary sequency) or translation inhibition (imperfectly complementary) (Fig. 1). MicroRNAs are mostly generated through the canonical pathway although some are generated through non-canonical pathways [4–6]. In the canonical pathway, a miRNA gene is transcribed by RNA polymerase II and further cleaved by microprocessors such as Drosha and DiGeorge Syndrome Critical Region 8 (DGCR8) within the nucleus to form a precursor miRNA (pre-miRNA). After exiting the nucleus by the help of nuclear protein Exportin 5, pre-miRNA is then processed by a ribonuclease called Dicer and results in a mature miRNA duplex. In order for miRNAs to bind to mRNA for gene silencing, Dicer is responsible of cleaving the miRNA duplex into two strands and one of them, referred as the guide strand, forms a miRNA-induced silencing complex (miRISC) by attaching onto Argonaute (AGO), which then guides the binding of the miRISC to the target mRNA sequence. The leftover strand is commonly named the passenger strand which is then degraded. Depending on which strand from the duplex is selected, either running from the 5’ side or the 3’ side, the miRNA is named with the notation ‘-5p’ or ‘-3p’ respectively. In some cases, both strands go on to form miRISCs, although studies have found that some -5p and -3p miRNAs exist in different abundances and regulate different mRNA targets, resulting in sometimes opposing physiological effects.

Some miRNAs are generated via a number of non-canonical pathways, one example is the primary miRNAs (pri-miRNAs) transcribed from the miRtrons (located within the intron region of protein coding genes) that cannot be cropped by Drosha and DGCR8 [4, 6]. Instead, these undergo a splicing process by debranching enzyme 1 (DBR1) which results in a shorter sequence. By studying the changes in mRNA expression in miRNA-transfected cells using microarray approaches, researchers have shown that multiple miRNAs can target the same mRNA, and one miRNA can regulate hundreds of targets, which directly affects the amount of proteins translated [7–11]. Therefore, many believe that miRNAs play a pivotal role in regulating numerous cellular processes resulting in significant physiological changes.

## miRNAs in cancer

While plenty of studies have demonstrated the role of overexpressed/suppressed expression of miRNAs in diseases such as cardiovascular diseases, autoimmunity and neurodegenerative disorders, cancer was the first disease ever proven to involve miRNAs during pathogenesis [12]. In 2002, Calin and colleagues discovered that both *miR-15* and *miR-16* genes are located on the frequently deleted chromosomal region in chronic B cell lymphocytic leukaemia [13]. They later found that both miR-15a and miR-16–1 directly downregulate BCL-2 expression and induce apoptosis [14]. With the advancement of miRNA profiling, not only has it been revealed that human miRNAs are frequently located at genomic sites that commonly exhibit DNA copy number abnormalities, and are consequently highly associated with cancer development, but it was also discovered that miRNA-associated genes such as *Dicer1* and *Agonaute2* have copy number alterations in several cancers including ovarian cancer [15–17]. A more recent study utilised previously generated whole genome sequencing datasets from The Cancer Genome Atlas (TCGA), and computational analysis to show that significantly over-mutated miRNA genes were commonly found across 33 different cancer types and associated with patient survival and cancer staging [18].

Since then, studies have discovered dysregulated miRNAs in almost all types of cancers, whether overexpressed or suppressed. These altered miRNA expressions are classified as either oncogenic (oncomiRs) or tumour suppressive miRNAs based on the affected downstream signalling pathways and the overall effect on disease progression. Examples of oncomiRs include miR-23b and miR-27b which both promote breast cancer cell proliferation and migration in vitro [19]. Implanting mice with breast cancer cells that had CRISPR-knockout of both miRNAs led to significantly reduced tumour volume and improved overall survival indicating an oncogenic role for both miR-23b and miR-27b in breast cancer. On the other hand, another study in which mice received xenograft transplantation of liver cancer cells that were transfected with miR-212-5p mimics, were later found to have significantly smaller tumours compared to control [20]. This was through decreased cancer cell proliferation and increased apoptosis via directly inhibiting the suppressor of cytokine signalling 5 (SOCS5). Interestingly, miRNAs can act as both tumour promoter and suppresser in different types of cancers. Overexpression of miR-424 was associated with worse survival outcome in pancreatic cancer [21], yet it was downregulated and suppressed proliferation in hepatocellular carcinoma [22]. To sum up, extensive in silico, in vitro and in vivo evidence indicate that miRNAs are heavily involved in regulating cell proliferation, growth, metabolism, and death, and by expanding our understanding of miRNA dysregulation in cancer, we will reveal more insights on tumour progression.

## Pancreatic ductal adenocarcinoma (PDAC)

Pancreatic cancer is well known for its extremely poor prognosis. Recent estimates by GLOBOCAN suggested that 495,773 patients globally were diagnosed with pancreatic cancer in 2020 and another 466,000 patients died as a result of pancreatic cancer [23]. Despite pancreatic cancer being ranked 13th in incidence amongst other cancer types, pancreatic cancer is projected to become the 2nd and 3rd leading cause of cancer-associated death in the United States and Europe respectively by 2030 [24, 25]. Over 90% cases of pancreatic malignancies are classified as PDAC, characterised by poor prognosis and survival. Vague clinical symptoms and late presentation result in over 80% of patients having metastatic disease at time of diagnosis. Once disseminated, surgical resection of the primary tumour is not usually recommended, and patients primarily rely on chemotherapy as a systematic treatment for prolonged survival.

Since the first study associated miRNAs with cancer development, investigating the role of miRNAs in different cancers has become an emerging area of research. In fact, increasing evidence continues to identify new miRNAs that are directly involved in driving the progression of PDAC [26]. In addition, extracellular miRNAs have been found to be transported in extracellular vesicles (EVs) that can reprogram both proximally and distally located cells. These EVs can be found present in a range of bodily fluids, due to their high stability, making them an excellent candidate biomarker for disease diagnosis, prognosis and even indication of treatment response. Studying the importance of miRNAs in PDAC will likely yield novel insights into the disease that cannot be uncovered by solely investigating individual proteins or pathways.

## Dysregulated miRNAs in pancreatic cancer

MicroRNAs are found to be dysregulated in cancer due to alterations on the biogenesis of miRNA in different stages, including modifications in chromosomes, epigenetics, transcriptional and microprocessor activity on pri-miRNA/pre-miRNA production, miRISC activity and interactions between miRNAs and other non-coding RNAs (Fig. 1). In the last decades, many studies have attempted to catalogue all of the dysregulated miRNAs in PDAC primary tumours, pancreatic juice [27] and blood [28–30] which will likely identify new miRNA and downstream pathway targets that can be exploited as novel treatments or diagnostic tools. We have summarised some examples of upregulated and downregulated miRNAs recently (within the last 5 years) discovered in human PDAC tumours, compared to adjacent normal pancreatic tissue, that were identified using miRNA sequencing/microarray (Tables 1 and 2).

**Table 1:** Recent examples of upregulated miRNA expression in human PDAC

miRNA	Targeted mRNA	Functional analysis	Associated signalling pathway/process	References
-21	ARHGAP24	Promotes proliferation and cell cycle progression, inhibits apoptosis in vitro	Predicted changes in Rho GTPase pathway [not experimentally validated]	[32]
	Spry2	Promotes tumour growth in vivo, enhances proliferation in vitro	Validated upregulation in MAPK/ERK, PI3K/Akt pathways	[33]
-30b-5p	GJA1	Promotes angiogenesis in hypoxic cancer cells in vitro and in vivo	Predicted changes in gap junction communication [not experimentally validated]	[34]
-135a/b	PFK1	Promotes cancer cell survival with glutamine deprivation in vitro and in vivo	Validated downregulation in aerobic glycolysis	[35]
-194-5p	SOCS2	Promotes cancer cell proliferation and migration in vitro and in vivo	Validated upregulation in PI3K/Akt pathway	[36]
-221, -222	TIMP-2	Promotes proliferation and invasion, enhances MMP-2 and -9 expression in vitro	Predicted changes in the extracellular matrix by altering MMP-2 and -9 [not experimentally validated]	[37]
-361-3p	DUSP2	Promotes EMT via inhibiting ERK1/2 pathway, enhances liver metastasis in vivo	Validated upregulation in ERK signalling	[38]
-501-3p	TGFBR3	Promotes cancer cell migration and invasion, metastasis in vitro and in vivo	Validated activation in TGF-β signalling	[39]
-608	BRD4	Promotes cancer cell apoptosis in vitro	Predicted changes in MYC-associated pathway [not experimentally validated]	[40]
-708-5p	SIRT3	Promotes cancer cell proliferation, migration, and invasion in vitro	Predicted changes in ROS-associated Src/FAK signalling [not experimentally validated]	[41]
-1469-5p	NDRG1	Promotes cancer cell proliferation and invasion in vitro	Validated downregulation in NF-kB signalling	[42]

*ARHGAP24* Rho GTPase Activating Protein 24; *Spry2* Sprouty RTK Signalling Antagonist 2; *GJA1* Gap Junction Protein Alpha 1; *PFK1* Phosphofructokinase-1; *SOCS2* Suppressor Of Cytokine Signalling 2; *TIMP-2* Tissue inhibitor of metalloproteinases 2; *MMP* Matrix metalloproteinases; *DUSP2* Dual Specificity Phosphatase 2; *TGFBR3* Transforming Growth Factor Beta Receptor 3; *BRD4* Bromodomain Containing 4; *SIRT3* Sirtuin 3; *ROS* Reactive oxygen species; *FAK* Focal adhesion kinase, *NDRG1* N-Myc Downstream Regulated 1

**Table 2:** Examples of downregulated miRNA expression in human PDAC

miRNA	Targeted mRNA	Functional analysis	Associated signalling pathway/process	References
-10b	E2F7	Enhances cancer cell proliferation, invasion, and migration in vitro	Predicted changes in cell cycle [not experimentally validated]	[43]
-15a	Wee1, Chk1, BMI-1, YAP-1	Promotes cancer cell proliferation and cell cycle progression in vitro	Predicted changes in cell cycle [not experimentally validated]	[44]
-24-3p	LAMB3	Promotes cell proliferation in vitro and tumour growth in vivo	Predicted changes in PI3K/Akt pathway [not experimentally validated]	[45]
	ASF1B	Promotes EMT, cell migration and invasion in vitro	Validated upregulation in VEGFA-associated pathway	[46]
-26a	E2F7	Promotes cell proliferation in vitro	Validated upregulation in VEGFA-associated pathway	[47]
-29	LOXL2	Promotes collagen crosslinking in vitro	Predicted changes in LOXL2-mediated collagen crosslinking [not experimentally validated]	[48]
-29b-2-5p	Cbl-b	Promotes cancer cell proliferation in *vitro* and in vivo	Validated overexpression in p53	[49]
-29c	MAPK1	Promotes cancer cell proliferation, migration and invasion in vitro and tumour growth in vivo	Validated inhibition in MAPK/ERK pathway	[50]
-30a-3p	ITGA2	Suppresses cell proliferation, migration, and invasion in vitro	Predicted changes in FAK pathway [not experimentally validated]	[51]
-30a-5p	FOXD1	Promotes cancer cell proliferation, cell cycle progression in vitro	Validated activation in ERK pathway	[52]
-30d	RUNX1	Promotes tumour growth, metastasis, and angiogenesis in vitro and in vivo	Validated activation in aerobic glycolysis	[53]
	SOX4	Promotes cancer cell proliferation and invasion in vitro and in vivo	Validated promotion in PI3K/Akt signalling	[54]
-33a-5p	RAP2A	Promotes cancer cell proliferation, migration, and invasion in vitro	Predicted changes in AKT signalling	[55]
-98-5p	MAP4K4	Promotes tumour growth by downregulating MAP4K4 in vitro and in vivo	Validated inhibition of MAPK/ERK pathway	[56]
-122-5p	CCNG1	Promotes EMT, cell proliferation, migration, and invasion in vitro	Predicted changes in cell cycle progression [not experimentally validated]	[57]
-143-3p	KRAS	Promotes cancer cell proliferation and migration in vitro and in vivo	Validated activation of ERK signalling	[58]
-193a-3p	CCND1	Promotes cell proliferation in vitro	Predicted changes in cell cycle progression [not experimentally validated]	[59]
-194-5p	PD-L1	Promotes EMT, proliferation, migration, and invasion in vitro, promotes tumour growth and suppresses CD8 T cell infiltration in the tumour in vivo	Validated downregulation in PD-1/PD-L1 pathway	[60]
-204-5p	RACGAP1	Promotes cell migration and invasion in vitro	Predicted changes in ERK and/or STAT3 signalling [not experimentally validated]	[61]
-323a-3p	HK-2	Promotes cancer cell proliferation in vitro, promotes tumour growth and metastasis in vivo	Predicted promotion in cancer cell glycolysis [not experimentally validated]	[62]
-340	CD47	Promotes tumour growth in vivo	Validated downregulated expression in inflammatory immune phenotype	[63, 64]
-345-5p	CCL8	Promotes cancer cell proliferation and migration in vitro and in vivo	Validated promotion in NF-kB pathway	[65]
-374b-5p	KDM5B	Promotes EMT, tumour growth and metastasis in vitro and in vivo	Validated promotion in EMT phenotype	[66]
-628-5p	PLSCR1, IRS1	Promotes cell proliferation, migration, and invasion in vitro	Validated upregulation in AKT/NF-kB pathway	[67]
-3662	HIF-1α	Promotes glycolysis in cancer cells and resistance to gemcitabine in vitro	Validated promotion in aerobic glycolysis	[68]

*E2F7* E2F Transcription Factor 7; *Chk1* Checkpoint kinase 1; *YAP-1* Yes-associated protein 1; *LAMB3* Laminin Subunit Beta 3; *ASF1B* anti-silencing function 1B; *VEGFA* Vascular endothelial growth factor A; *LOXL2* Lysyl Oxidase Like 2; *Cbl-b* Cbl Proto-Oncogene B; *MAPK1* Mitogen-activated protein kinase 1; *ITGA2* Integrin Subunit Alpha 2; *STAT3* Signal transducer and activator of transcription 3; *FOXD1* Forkhead Box D1; *RUNX1* Runt-related transcription factor 1; *SOX4* SRY-Box Transcription Factor 4; *RAP2A* Ras-related protein 2A; *CCNG1* Cyclin G1; *EMT* Epithelial–mesenchymal transition; CCND1 Cyclin D1; *PD-L1* Programmed death-ligand 1; *RACGAP1* Rac GTPase Activating Protein 1; *HK-2* Hexokinase 2; *CCL8* C–C Motif Chemokine Ligand 8; *KDM5B* Lysine Demethylase 5B; *PLSCR1* Phospholipid Scramblase 1; *IRS1* Insulin Receptor Substrate 1; *HIF-1α* Hypoxia Inducible Factor 1 Subunit Alpha

To date, studies have commonly utilised publicly available software/databases such as TargetScan, miRbase, miRDB and miRTarBase to predict which mRNAs that a specific miRNA is likely to bind to based on their nucleotide sequences [31]. These are then typically verified experimentally by conducting a luciferase based-reporter assay which measures the binding activity between the 3’UTR of the target mRNA and the miRNA, compared to a mutated control. Functional assays are then performed to examine the biological relevance of miRNA expression on cell proliferation, migration, invasion, and metastasis in vitro and in vivo using cell lines and/or murine implantations.

## Genetic alterations and the interplay with miRNAs in PDAC initiation and progression

**Fig. 2:**
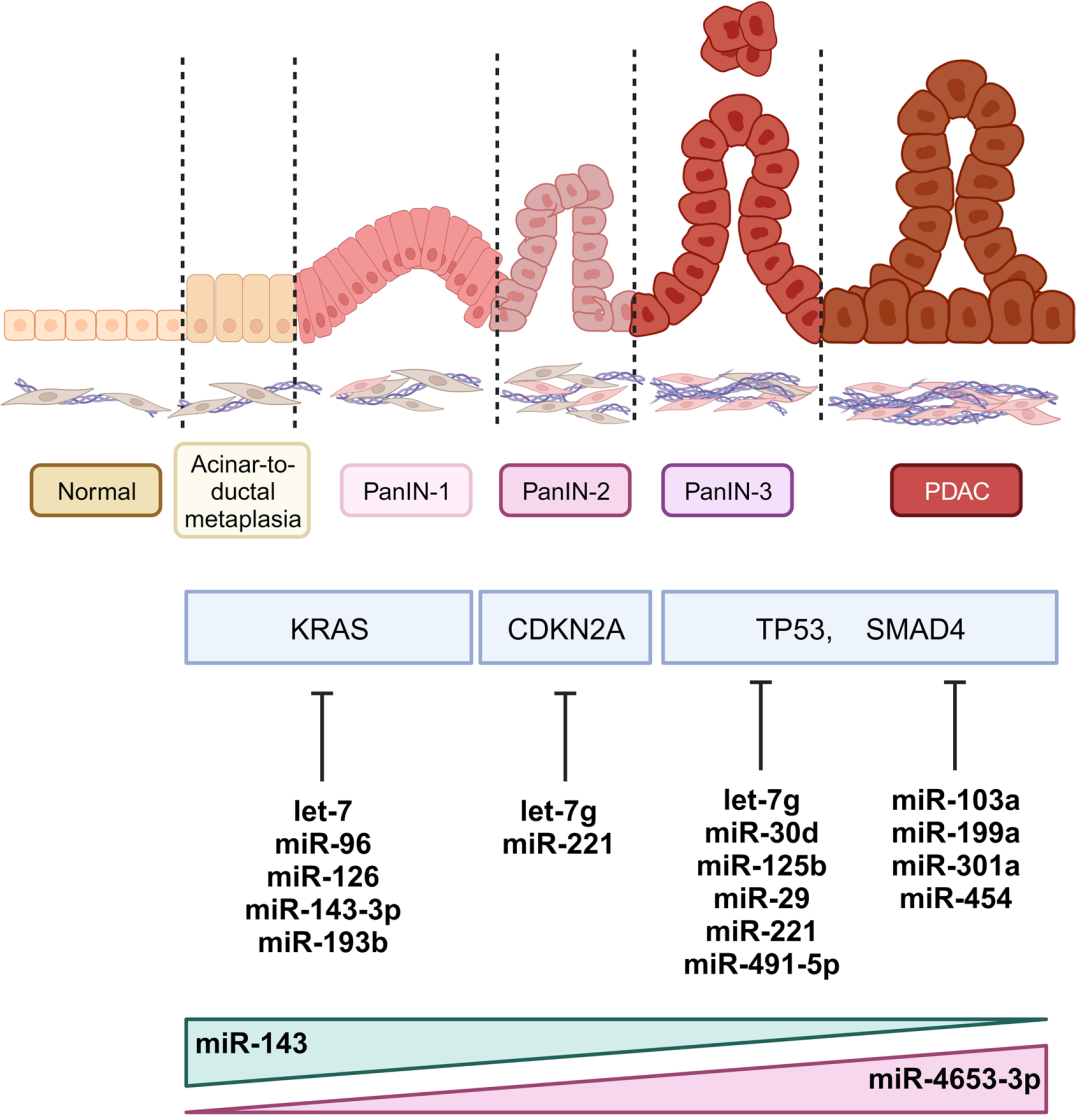
The progression of PDAC is often associated with mutations of *KRAS*, *CDKN2A*, *TP53* and *SMAD4* expressions, which can be directly modulated by several miRNAs. Certain miRNAs are also found to be increased/decreased in expression during different stages of PDAC development

Many risk factors are associated with the development of PDAC and one of the biggest contributors to the initiation of PDAC is genetic alterations [69]. Although it varies between patients, the carcinogenesis of PDAC is a highly multifaceted process that involves the interplay of many molecular, cellular, and acellular elements. The development of full-blown PDAC is preceded by three stages of morphologically distinct intraepithelial lesions (PanIN) that arise from normal pancreatic acinar cells. Major genetic mutations that are found across different stages of PanIN include *KRAS* mutations in the early stage of PanIN-1, loss of *CDKN2A* across PanIN-1 to PanIN-3, loss of *SMAD4* and *TP53* in PanIN-3 leading to PDAC [70] (Fig. 2).

In recent years, several miRNAs have been demonstrated to directly regulate and/or interact with these commonly mutated genes which suggests a strong link between miRNA and PDAC initiation and progression [58, 71]. For example, miR-193b levels are reduced in PDAC and in the early epithelial changes around the tumour tissue as compared to adjacent normal tissue [72]. Furthermore, both miR-193b and miR-143-3p can directly regulate KRAS, which promoted cancer cell growth in vitro and in vivo [58, 71]. Further evidence indicates that the absence of miR-802 in both the KC (*Ptf1a*^Cre/+^ *Kras*^G12D/+^) and KPC (*Ptf1a*^Cre/+^ *Kras*^G12D/+^ *P53*^R172H/+^) mouse model led to a striking increase in acinar-to-ductal lesions in mouse models, but showed an insignificant effect on human PDAC cancer cell proliferation, indicting miR-802 may be particularly fundamental in regulating the initiation of PDAC [73].

In addition, dysregulated miRNA expression, mediated through epigenetic changes and changes in miRNA biogenesis, can subsequently result in altered mRNA expression of oncogenes [74]. One of the key enzymes in the biogenesis of miRNAs is Dicer. A recent study uncovered Dicer expression is not only upregulated in advanced PDAC tissue, but it also controls metabolic changes in cancer cells that promote cell growth and resistance to gemcitabine chemotherapy [75].

To further demonstrate the role of miRNAs in PDAC development, multiple studies have shown certain cancer-associated miRNAs are differentially expressed throughout malignant progression. Utilising immunohistochemistry coupled with in situ hybridisation, miR-143 expression levels were found to decrease over the stages of PanIN 2, 3 and PDAC [76]. Functional studies verified that higher expression of miR-143 suppressed cancer cell proliferation and invasion in vitro via directly regulating mucin 13 (MUC13). Another study from Hirabayashi et al. identified that miR-4653-3p is progressively increased from early to late PanIN and PDAC, and not expressed in normal pancreatic tissue [77], HIPK2 was found to be a direct target of miR-4653 and its pattern of expression is inverse to miR-4653. Since HIPK2 has been previously reported to regulate the tumour suppressor gene *p53*, it is hypothesised that increased miR-4653 in later stages of PDAC suppresses the expression of HIPK2 thereby contributing to poor prognosis.

Despite the evidence of dysregulated miRNAs in modulating mRNA expression, it is noteworthy that miRNAs are not always the initiator of these changes. miRNAs can be modulated by other endogenous non-coding RNAs such as long non-coding RNA (lncRNA) and circular RNA (circRNA) which also affect cancer progression at a post-transcriptional level. Increasing evidence indicates that these other non-coding RNAs compete to bind to miRNAs and act as a ‘sponge’ to inhibit the downstream mRNA regulatory activity [78, 79].

## miRNA effects on cell proliferation and cell cycle progression

Downstream of oncogenic KRAS are two key signalling pathways; the MAPK/MEK/ERK and PI3K/Akt cascades; which are critical to cell proliferation, cell cycle progression and survival [80]. Several miRNAs have been suggested to regulate the expression of oncogenes or tumour suppressing genes which ultimately either positively or negatively contribute towards tumour development [81]. For example, downregulated miR-29c was found in PDAC tissues and was associated with upregulated MAPK1 and resulted in increased cell proliferation, and invasion through activating the downstream MAPK/ERK pathway [50]. Similarly, downregulated expression of miR-98-5p was shown to lead to upregulated MAP4K4 which promoted cell proliferation, invasion, and migration of PDAC cells in vitro by promoting MAPK/ERK signalling [56].

**Fig. 3:**
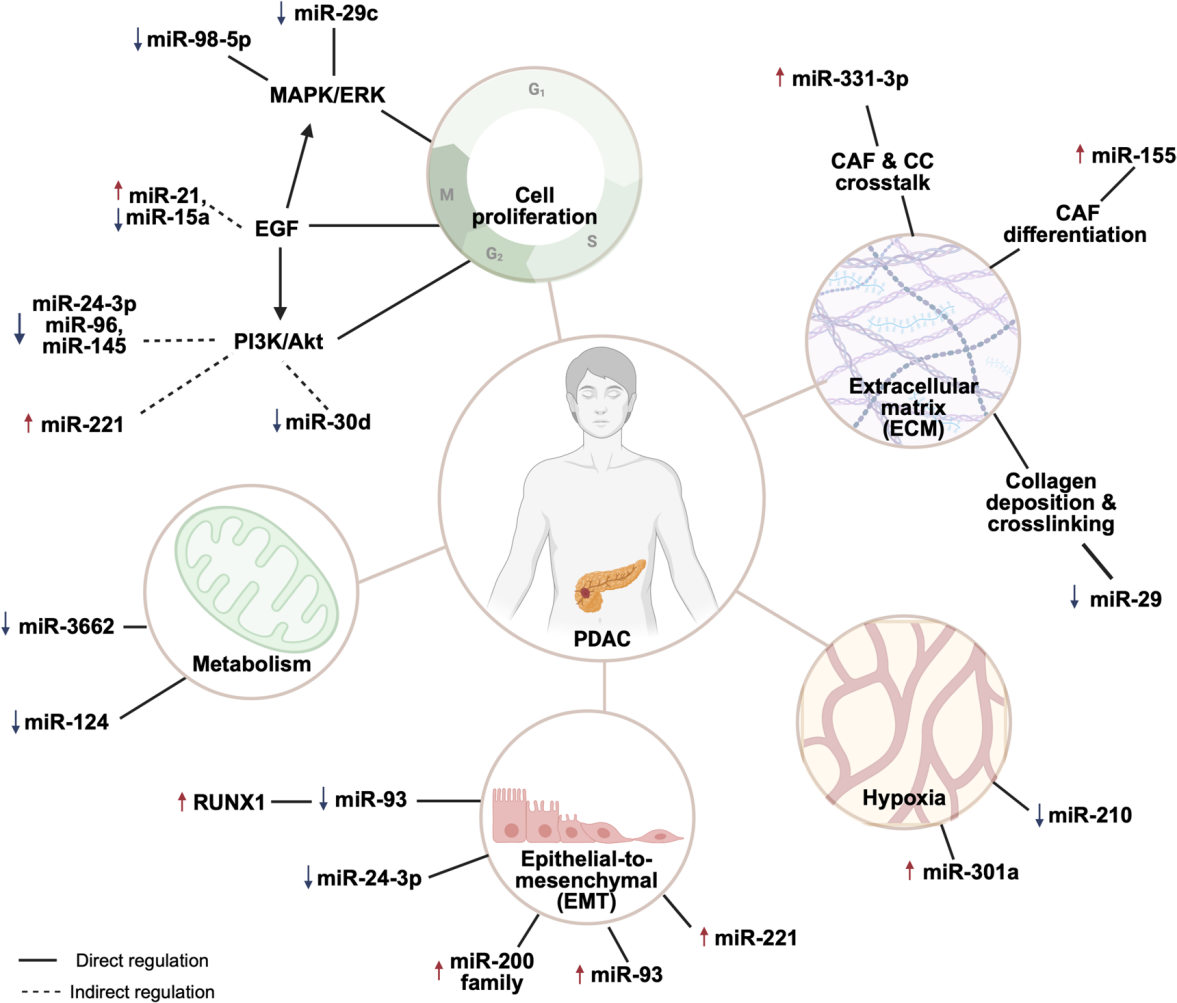
Examples of miRNAs involved in PDAC progression

In another study, miR-21 was found to stimulate the EGF pathway through binding to the sprouty RTK signalling antagonist 2 (Spry2), resulting in increased cell proliferation and activation of downstream MAPK/ERK and PI3K/Akt signalling pathways [33]. Many miRNAs can have multiple mRNA targets, one example being miR-24-3p, which can target both Laminin Subunit Beta 3 (LAMB3) and anti-silencing function 1B (ASF1B), contributing to the aggressive nature of PDAC by activating PI3K/Akt pathway and triggering EMT respectively [45, 46]. In 2021, two separate groups found that low miR-30d expression is associated with poor prognosis in PDAC, and that this miRNA can target RUNX1 and SOX4 to promote cancer cell malignancy [53, 54]. Interestingly, a previous study had discovered that RUNX1 can negatively regulate miR-93 through binding to its promoter region, which in turns inhibits EMT, invasion and migration in PDAC cells [82]. This illustrates how complex the miRNA/mRNA regulatory networks are, even without considering the interference of other non-coding RNAs. As such, even small changes in miRNA levels can lead to significant shifts in the equilibrium of these regulatory networks with direct impacts on downstream cellular phenotype (Fig. 3).

## miRNA roles in PDAC metabolism

One distinctive phenotype of cancer cells is dysregulated metabolism. Cancer cells can generate large amounts of energy through increased uptake and metabolism of glucose via anaerobic rather than aerobic glycolysis, even under normoxia (termed the 'Warburg effect') [70]. Metabolism is critical to cell proliferation, migration, and survival, and in PDAC, the Warburg effect has been linked to increased resistance to chemotherapy [83]. MicroRNAs have been implicated in various facets of cancer cell metabolism. For example, miR-3662 has been shown to decrease the Warburg effect by targeting glycolytic genes including glucose transporter: solute carrier family 2 member 1 (SLC2A1) and glycolytic enzymes: phosphofructokinase platelet (PFKP), pyruvate kinase M (PKM) and lactate dehydrogenase A (LDHA) [68].

Furthermore, in a gemcitabine-resistant murine xenograft model, it was demonstrated that overexpression of miR-3662 could re-sensitise the tumour to gemcitabine, resulting in reduced tumour growth via directly reducing levels of hypoxia-inducible factor (HIF-1α), which is known to promote glycolysis in cancer [83]. Meanwhile, another study has shown miR-124 regulates monocarboxylate transporter 1 (MCT1), a downstream target of HIF-1α [84]. Inhibiting lactate transporting MCT1 results in changes in cell acidity and inhibits cancer cell proliferation and invasion in vitro and in vivo. Although increasing number of studies have been performed in recent decades to explore the role of miRNA in a range of cancers, limited research has been conducted in PDAC. Considering the importance of cancer metabolism, further research should be performed which may shed light into novel therapeutic interventions targeting the aberrant metabolic pathway.

## miRNAs in epithelial mesenchymal transition (EMT) and hypoxia

The term EMT refers to the process by which cancer cells with an epithelial phenotype adopt a more mesenchymal phenotype, which is known to associate with their ability to invade, migrate and even become resistance to chemotherapy-induced cell death. EMT-associated genes have also been highly related to disease prognosis, and studies have uncovered several miRNAs that are thought to directly regulate EMT genes. The miR-200 family (miR-200a, miR-200b, miR-200c, miR-124, miR-429) well-known for its ability to maintain a cancer cell epithelial status and prevent EMT. For example, miR-200b and miR-200c expression has been correlated with the formation of tumour budding, a classic EMT feature. Although their expression levels vary between studies, the miR-200 family is typically upregulated in PDAC tumours compared to matched normal pancreas. Upregulation of miR-200 family members typically results in a suppression of the EMT-inducing zinc finger E-Box-Binding homeobox (ZEB) family. This in turn releases the ZEB repression of E-cadherin expression. E-cadherin is a key cadherin subtype associated with the maintenance of an epithelial phenotype [85–87]. This effect has been confirmed in cells that were induced to undergo TGF-β-mediated EMT, where there was a significant decrease in miR-200 family expression, which was accompanied by a gain in expression mesenchymal markers such as ZEB [88]. The association of these miRNAs with EMT markers have been further confirmed in a set of PDAC tumour tissues and sera [89]. A recent study by Huang et al*.* (2023) indicated that miR-24-3p directly targets ASF1B to subsequently promote an EMT phenotype in cancer cells resulting in enhanced invasiveness in vitro [46]. Additionally, overexpression of miR-24-3p using miRNA mimics significantly reduced cancer cell growth although further in vivo studies are required to further investigate miR-24-3p’s therapeutic potential.

Another salient feature of the tumour microenvironment is the limited oxygen supply, due to rapid depletion of oxygen by proliferating cancer cells, and/or compressed/blocked or otherwise insufficient vasculature. Increases in hypoxia within the tumour microenvironment have been shown to drive the progression of EMT in PDAC [90]. Overexpression of miR-301a promotes the adoption of a more mesenchymal phenotype and upregulation of HIF-1α in through directly targeting TP63 [91]. In an oxygen-deficient environment, PDAC cells were also shown to release more exosomal miR-30b-5p which subsequently promotes angiogenesis by inhibiting gap junction protein (GJA1) in endothelial cells. Similar studies have shown that cancer cells that produce more miR-210 in exosomes may also promote EMT, increased cellular permeability and enhanced tumour vascularisation [92, 93].

## miRNA regulation of extracellular matrix (ECM) remodelling and stromal cell behaviour

A key characteristic of PDAC is the extensive desmoplasia found in and around the tumour. This desmoplastic response increases over the course of PanIN and PDAC development, typically causing a physical barrier to treatment as well as providing a route for cancer cells to metastasise [94]. Therefore, increasing efforts are being made to understand and target the stroma in PDAC, in combination with conventional, already approved therapies to increase treatment efficacy.

A key contributor to, and regulator of the ECM is the cancer-associated fibroblasts (CAFs), which is responsible for producing a large proportion of ECM components. As such CAFs and CAF-mediated matrix deposition and remodelling are often considered tumour promoting. In the recent decade, some studies have suggested that cells in the tumour microenvironment utilise EVs containing miRNAs to communicate with other cells, and thereby influence their cellular phenotypes [95, 96]. In 2015, Pang and colleagues were the first to demonstrate that PDAC cells may promote the differentiation of CAFs/CAF-like cells from normal fibroblasts through the secretion of miR-155 containing EVs, which can directly bind to TP53INP1 [97]. The conversion of CAFs was confirmed by measuring the protein level of TP53INP1, the CAF markers α-SMA and fibroblast activation protein (FAP) in fibroblasts that were co-cultured with PDAC cell lines or media containing PDAC cell-derived EVs. On the other hand, CAFs have also been shown to reciprocally influence cancer cells through miRNA secretion. In this recent study, cancer cells co-cultured with CAF-derived EVs containing miR-331-3p promoted the proliferation, migration, and invasion of cancer cells in vitro, potentially via directly inhibiting Scavenger Receptor Class A Member 5 (SCARA5) [98]. Other previous studies have demonstrated that suppressing SCARA5 expression resulted in accelerated tumour progression by activating FAK signalling [99] as well as promoting Snail1-regulated EMT and cancer cell migration [100].

MicroRNAs have also been shown to play a role in regulating the secretion of matrix and matrix modifying components. Lui et al. reported earlier this year that differentially expressed miRNAs in PDAC patients are highly associated with ECM organisation and remodelling [81]. In this study, serum miRNAs were sequenced and found to be associated with important cancer-driven genes, such as *KRAS*, and other ECM-related genes such as matrix metalloprotease 14 (*MMP14*), plasminogen activator urokinase (*PLAU*) and tenascin C (*TNC*). Moreover, *KRAS* activation was found to associated with the ablation of miR-29 expression in PDAC, resulting in increased deposition of ECM proteins by CAFs and the promotion of cancer cell colony formation in vitro [101]. From the same miRNA family, miR-29a has been shown to inhibit the transcription of lysyl oxidase like 2 (LOXL2), an important ECM enzyme that is responsible for collagen crosslinking and promoting EMT in PDAC [48]. Another study has demonstrated the importance of miR-21 in early development of PDAC, specifically in regulating CAF phenotype and function, as well as modulating the phenotype of immune cell populations within the tumour microenvironment. However in this work, there were no significant effects on cancer cells which indicates that some miRNAs may play specific roles in only certain cell types [102]. Nevertheless, there is currently a lack of understanding regarding miRNAs that specifically regulate the matrisome of PDAC. This is in part because most miRNA sequencing results from PDAC tumours were performed on bulk tumour pieces, and as such ignore the heterogeneity of the tumour ecosystem, making it difficult to assign specific miRNA profiles to the stromal proportion of the PDAC tumour. In future, further studies are needed to understand miRNA’s role specifically in regulating the deposition, organisation and remodelling of the ECM.

## The role of miRNA in pancreatic cancer metastasis

**Fig. 4:**
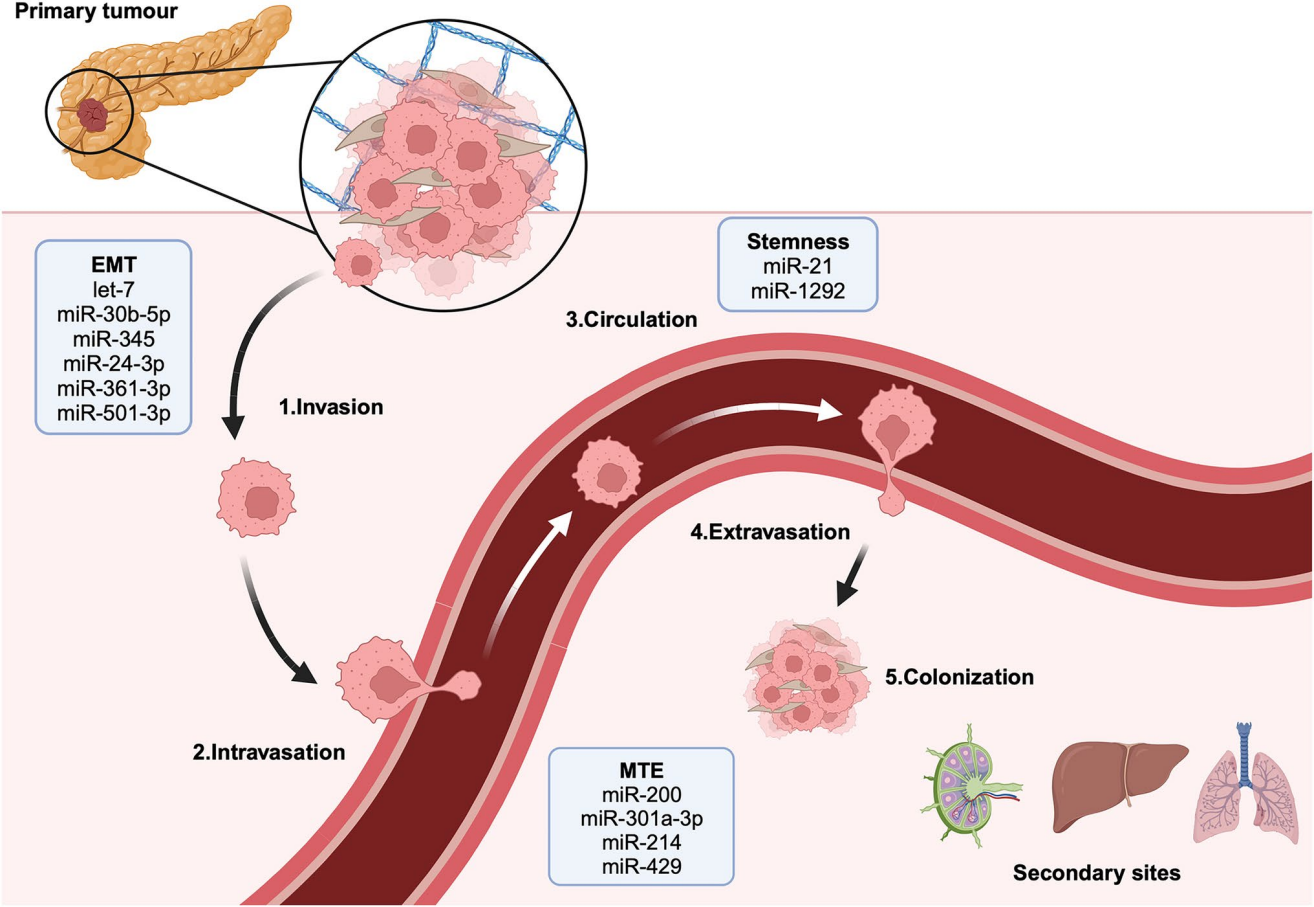
miRNAs have been shown to be implicated in multiple steps of the metastatic cascade in PDAC including: 1. Cancer cell migration and invasion at the primary tumour 2. Intravasation into the blood and lymphatic systems 3. Survival and transit in the circulation 4. Extravasation from the vessels and invasion at the secondary sites 5. Colonisation and expansion at the secondary sites

The poor prognosis PDAC patients face is not only due to difficulties in treating the primary tumour but also in treating disseminated cancer cells that have colonised other organs, such as the liver. Metastasis is a multi-step process that involves a multitude of cell types, and an environment that favours the establishment of secondary tumours (Fig. 4). Cancer cells that successfully invade into the local tissue microenvironment surrounding primary tumours, and breach the vascular basement membrane (intravasation), can then enter the circulation, and disseminate around the body. At some point, usually within a secondary tissue, cancer cells then extravasate (exit the circulation) and begin colonisation of the secondary site. At this stage they may also become dormant.

During the initial stage of local invasion, miRNAs play a role in aiding migration by promoting EMT [103, 104]. An example miRNA in EMT promotion is miR-361-3p, that directly targets dual-specificity phosphatase–2 (DUSP2) to activate ERK signalling pathway and promote EMT, resulting in increased cancer cell invasion and migration in vitro [38]. In vivo miR-361-3p overexpression results in an increased number of metastatic nodules in the liver in mice.

Tumour vasculature is thought to provide a critical route of metastasis to secondary sites. It has been shown that under oxygen-deficient conditions, pancreatic cancer cells produce more EVs that are enriched for miR-30b-5p [34] that trigger angiogenesis. Evidence in support of this was gained when endothelial cells transfected with miR-30b-5p mimics exhibited a higher total tube formation length and increased in migration ability possibly through the miR-30b-5p inhibition of GJA1.

Tumour-associated macrophages (TAMs) have been associated with promoting invasion and angiogenesis in solid cancers, and previous evidence has demonstrated that miRNA-containing vesicles can be produced by both TAMs and cancer cells to modulate each other’s tumour-promoting behaviour. For example, hypoxic cancer cell-derived miR-301a induces a macrophage M2 polarisation via the PTEN/PI3Kγ pathway. Meanwhile, M2 polarised macrophages have been shown to promote cancer cell EMT, invasion and metastasis [105]. At the same time, M2 macrophages secrete miR-501-3p that has been shown to promote cancer cells growth and metastasis in vitro and in vivo via inhibiting anti-tumourigenic TGF-β Receptor III (TGFBR3) [39].

In recent years, it has become evident that distant secondary sites may be primed in advance by tumours, in a process which was coined the pre-metastatic niche. There is clear evidence that cancer cells secrete miRNA-containing EVs as a form of cell-to-cell communication, and recent evidence has also implicated these EVs in pre-conditioning metastatic sites in certain tumours [106, 107]. In addition, metastasising mesenchymal-like cancer cells have been observed to undergo mesenchymal-to-epithelial transition (MET) and revert to a more epithelial phenotype which is considered to be beneficial for secondary site colonisation. As discussed above, the miR-200 family has been well studied for their regulatory activity in promoting an epithelial phenotype through reducing the activities of ZEB1 and ZEB2 [86, 108]. In particular overexpressed miR-429, a direct regulator of ZEB1, has been shown to promote the adoption of an epithelial phenotype in cancer cells in secondary sites [86].

To date, there are no clinically approved drugs that specifically target the metastasis process in cancer, despite modern advancements in drug discovery. This is largely due to the complexity of the metastatic cascade. Given the importance of miRNA regulatory activities, the targeting of dysregulated miRNAs in metastasis could allow a combined effect on multiple tumour-promoting mRNAs. Furthermore, miRNAs can contribute to improve on our current diagnostic and prognostic prediction. By quantifying circulating serum levels of miR-607 in a cohort of 184 PDAC patients, it was found that miR-607 was significantly correlated with the presence of lymphatic and liver metastasis, overall survival, and progression-free survival, supporting miRNA’s potential as diagnostic and prognostic biomarkers [29]. Therefore, monitoring the expression of cancer-associated miRNAs involved in the various stages of the metastatic cascade could potentially indicate disease progression. Furthermore, novel therapeutic approaches could be developed to target these miRNAs with either miRNA mimics/inhibitors to modulate key downstream signalling pathways. For instance, miR-210 has been associated with pancreatic cancer cell proliferation, invasion, and metastasis and in a cohort of PDAC mice, treatment with a miR-210 inhibitor demonstrated improved survival and reduced liver metastasis compared to the control [109].

## miRNAs and resistance to pancreatic cancer treatment

### Chemotherapy

With many patients presenting in the clinic with already disseminated disease that is surgically unresectable, systemic chemotherapy, sometimes in combination with radiotherapy, remains the mainstay of treatment. The current recommended first-line therapy for PDAC is either FOLFIRINOX (a combination regimen of oxaliplatin, leucovorin, irinotecan and 5-fluorouracil) or gemcitabine plus nab-paclitaxel [110]. However, the desmoplastic response in PDAC can create a physical barrier for drug delivery, and in combination with other cellular factors can lead to the emergence of resistance. Acquired resistance often arises from a change at a genetic level (such as acquiring a new mutation), however changes in expression and/or activity of one transcriptional/translational regulator may also lead to the emergence of resistance. One such regulator has been shown to be miRNAs (Table 3).

**Table 3:** Recent evidence of miRNAs that can be used to sensitise PDAC patients against chemotherapy

miRNA	Intervention	Target mRNA	Chemotherapy	References
-342-3p	Inhibition	KLF6	Gemcitabine	[117]
-1307	Inhibition	CLIC5	FOLFIRINOX	[116]
-30a-5p	Overexpression	FOXD1	Gemcitabine	[52]
-30a	Overexpression	SNA1	Gemcitabine	[115]
-3662	Overexpression	HIF-1α	Gemcitabine	[68]
-125a-3p	Overexpression	Fyn	Gemcitabine	[118]

*KLF6* Krüppel-like factor 6, *CLIC5* chloride intracellular channel 5, *FOXD1* forkhead box D1, *SNA1* Snail Family Transcriptional Repressor 1, *HIF-1α* hypoxia-inducible factor 1-alpha

Many studies have suggested the role of miR-155 in development of PDAC chemoresistance [111, 112]. Not only has it been shown that overexpression of miR-155 is associated with worse prognosis, but evidence has also indicated that cancer cells secrete exosomal miR-155 to pass on this resistant phenotype to neighbouring cells. Additionally, CAFs are also able to secrete exosomes to promote cancer cell growth and resistance to chemotherapy, and most surprising was the discovery that gemcitabine can promote miR-155 secretion from CAFs to create a positive feedback loop [113, 114]. Two studies in 2019 separately found that overexpression of the miR-30 family sensitises pancreatic cancer cells to gemcitabine through reducing two different genes: snail family transcriptional repressor 1 (SNAI1) and forkhead box D1 (FOXD1) [52, 115]. Other examples include the finding that abolished miR-1307 expression in pancreatic cancer cells led to an increase in DNA damage when exposed to FOLFIRINOX therapy [116]. As we develop a deeper understanding of the role that miRNAs play in therapy resistance, we can utilise this information to target key miRNAs with miRNA mimics/inhibitors to subsequently increase the efficacy of chemotherapy, as well as exploit them to monitor and even predict chemotherapy efficacy.

### Radiotherapy

The use of radiotherapy in PDAC also faces the challenge of developing resistance. Radiation therapy induces DNA damage and miRNAs have been found to be modulate signalling pathways that are crucial for cell cycle progression, DNA damage repair and apoptosis [119, 120]. MicroRNAs that have been associated with radiotherapy resistance in different cancers have been well summarised in [121], including work that has investigated the changes in miRNA expression in response to DNA damage caused by radiation in vitro*,* as well as providing the first in vivo evidence on circulating miRNAs from radiation-treated mice. Regardless, there has been limited exploration in the PDAC space to date.

In PDAC, ionizing radiation has been shown to be associated with upregulation of mammalian target of rapamycin (mTOR) activity via downregulating the mTOR-regulator miR-99, leading to increased pancreatic cancer cell survival and resistance to radiation [122]. More recently, radiation was found to upregulate miR-193-5p levels in PDAC cells in vitro which subsequently inhibited zinc finger protein 57 (ZFP57) and thus activated the WNT pathway, promoting resistance to radiation-induced cell death [123]. Radiation has also been demonstrated to promote EV secretion in cancer cells and a recent study found that radiation-exposed pancreatic cancer cells increased production of miR-194-5p containing EVs, which led to increased tumour repopulation for radiation [124]. However, the limitation of in vitro studies should be taken into consideration while reviewing the role of miRNAs in radiation resistance, since irradiating cell lines in culture does not fully recapitulate human patients in terms of radiation dosage, 3D tumour organisation and heterogeneity. The current dogma on the association between miRNA and radiotherapy resistance can only be verified by further research particularly in measuring patient samples. In addition, previous studies have shown that serum miRNA expression levels are modified post-radiotherapy in human patients of breast and prostate cancer [125, 126]. Similar characterisation of serum miRNA changes in PDAC patients before and after radiation exposure as well as correlating those to prognosis will further reveal the potential of utilising miRNA as an indication of radiotherapy efficacy.

## miRNAs as diagnostic and prognostic biomarkers for pancreatic cancer

### Diagnostic value

The 5-year overall survival rate for localised stage I PDAC is around 80%, but as soon as the tumour has spread to lymph nodes or to distant sites the survival rate significantly drops to 3.2% and 2.8% respectively [127]. Considering the generally poor prognosis of PDAC, the key to improving outcome is to diagnose patients at earlier stage while surgical resection is still feasible. Unlike some other cancers such as breast and colorectal cancer, where non-invasive routine checks for the general population have been implemented, examining a biopsy tissue remains the gold standard for diagnosing and staging PDAC. However, biopsy is mostly only obtained when patients become symptomatic and as a result, over 80% of these patients are diagnosed with metastatic PDAC at this point. Currently, the only routinely used blood biomarker is elevated carbohydrate antigen (CA) 19–9, which is not specific to PDAC and is sometimes an indication of non-malignant conditions, such as chronic pancreatitis (CP) and diabetes mellitus. In PDAC, CA19-9 typically has low sensitivity and specificity of 80% and 75% respectively [128]. In addition, CA19-9 has little value in diagnosing asymptomatic PDAC patients as 15–25% patients with pancreatic cancer also have low CA19-9 level [129].

Therefore, one major focus on PDAC research is discovering new biomarkers that are present in easily accessible samples, such as blood and other bodily fluids which are easier to obtain, and have the potential as diagnostic, prognostic and even surveillance biomarkers to screen for early-stage pancreatic malignancy. In recent years, the discovery of tumour produced miRNA-containing EVs into the circulation has generated much excitement [93]. The lipid bilayer of exosomes protects miRNAs against enzymatic degradation and exosomes have been shown to be extremely stable at 4˚C, -20˚C and -80˚C, making them an excellent biomarker candidate [130]. Furthermore, miRNAs in EVs are also known to be stable at 37˚C [131]. A protocol optimised by Dittmar et al. only required 20 µL plasma to collect around 68 different miRNAs, suggesting that miRNA detection as a biomarker in human plasma could be performed on small quantities and more importantly, in high throughput [96]. We have summarised some of the recent work on miRNA as PDAC biomarkers in a range of human bodily fluids (Table 4). To improve the overall diagnostic accuracy, several studies also investigated the sensitivity and specificity of miRNA biomarkers in combination with CA19-9.

**Table 4:** miRNA biomarkers detected in body fluids from PDAC patients

Body fluids	miRNAs	Sample size	Function	References
Plasma	-222	73	Correlate to tumour size, differentiation and TNM stage in PDAC	[137]
	-93-5p, -339-3p, 425-5p, 425-3p	34	The panel identified more early stage PDAC sample (80%) than CA19-9 (20%)	[28]
	-125a-3p, -4530, -92a-2-5p	142	Differentiate between PDAC from healthy control	[138]
	-95-3p/26b-5p	90	The ratio of miR-95-3p/miR-26b-5p can differentiate between PDAC from CP patients	[139]
	Panel of 13 miRNAs	292	Differentiate between PDAC and healthy control, particularly early stage (stage I and II) PDAC outperforming CA19-9	[129]
	-34-5p	88	Differentiate between PDAC from healthy control	[140]
	-130a-3p			
	-222-3p			
	-222-3p, -221-3p	66	Differentiate between PDAC from healthy control	[141]
Serum	-200b	89	Differentiate between PDAC from healthy control and CP patients	[142]
	-200c		Differentiate between PDAC from healthy control, but not from CP	
	-483-3p	85	Distinguish early stage PDAC sample from healthy control. miR-483-3p expression level is also correlated to PanIN grade in tissues	[143]
	-607	368	Correlated to lymph node and liver metastasis, perineural invasion, overall survival, and progression-free survival	[29]
	-215-5p, -122-5p, -192-5p, -30b-5p, -320b	125	Differentiate between PDAC from healthy control and CP patients	[30]
	-210-3p	77	Differentiate between PDAC from healthy control and CP patients. The expression level is correlated with CRP level and CA19-9	[144]
	-141, -200b, -200c	27	Differentiate between PDAC and non-PDAC (healthy control and CP patients)	[89]
	-6821-5p	24	Independently correlated to early PDAC with a better AUC than serum CA19-9	[135]
	-574-3p, -1202, -4466, -6831-5p, -6089	15	Differentiate between early PDAC patients from healthy control better than serum CA19-9	
	-4669		Differentiate PDAC patients with lymph node metastasis from patients without and healthy control	
Pancreatic juice	-21, -25, -16	172	The panel of pancreatic juice miRNAs and serum CA19-9 improved the differentiation between PDAC and healthy control	[27]
	-21	35	Differentiate between PDAC and CP patients better than serum CA19-9	[123]
	-155			
Peritoneal washing	-194-5p	59	Associated with peritoneal recurrence	[145]
Urine	-3940-5p/-8069	80	Differentiate between PDAC and CP patients, higher concentration in urine than in serum	[146]

*CP* chronic pancreatitis, *AUC* area under the ROC curve, *CRP* C reaction protein

While CA19-9 and clinical symptoms cannot reliably differentiate between different pancreatic disorders, studies revealed that there are tumour/serum miRNAs specific to PDAC which can aid accurate and non-invasive diagnosis [26, 132]. A study by Makler elucidated the tumour miRNAs (miR-31, -210, -339, -429, -1208) that are differentially expressed between chronic pancreatitis (CP) and PDAC, which is clinically valuable to ensuring a correct diagnosis [133]. They also discovered 18 altered miRNAs that are expressed in different stages of PDAC, which would also provide important information with regard to PDAC progression [133]. On the other hand, by analysing a total of 125 serum samples from healthy control or patients with either PDAC or CP, the authors found that 5 miRNAs (miR-215-5p, -122-5p, -192-5p, -30b-5p, -320b) were able to distinguish PDAC patients from non-PDAC individuals with CP and otherwise healthy, which is clinically valuable to ensuring a correct diagnosis particularly in CP patients who often have similar symptoms [25]. Besides CP and PDAC, another study has identified the differentially expressed serum miRNA profiles from other pancreatic lesions or neoplasms, such as pancreatic neuroendocrine tumour, intraductal papillary mucinous neoplasms and ampulla of Vater carcinoma, which could also be used in the clinic to assist diagnosis with further validation [134]. Six other serum miRNAs (miR-574-3p, -1202, -4466, -6831-5p and -6089) were found to independently differentiate early PDAC patients from healthy volunteers with a better area under the ROC curve (AUC) than that of serum CA19-9, indicating miRNAs are highly valuable in diagnosing patients in early stage PDAC, which coupled with intervention would lead to a higher chance of survival [135]. Finally, a recent study from Nakamura et al*.* optimised the use of 13 serum miRNAs, which could differentiate all stages PDAC from healthy controls, and more remarkably achieved excellent ability in diagnosing early stage PDAC (AUC: 0.93; sensitivity: 80%; specificity: 91%) [129]. The combination of this panel of miRNAs and serum CA19-9 level, was a superior diagnostic tool for early PDAC (AUC: 0.99; sensitivity: 93%; specificity: 93%), and presents a promising diagnostic approach for early detection of PDAC.

In addition to examining biomarkers in blood through liquid biopsy, research has also focussed on other bodily fluids that may contain tumour-specific miRNAs. For example, pancreatic juice collected from the duodenum during endoscope ultrasound (EUS) is thought to contain more miRNAs derived from the pancreas/tumour since it is produced by ductular cells in the pancreas, whereas plasma/serum miRNAs may be derived from other organs. The first study that investigated exosomal miRNAs in pancreatic juice was from Nakamura and colleagues in 2019. Although it only included 35 samples, they found that miR-21 and miR-155 can be used to identify PDAC patients from CP patients. The accuracy of diagnosis was further increased when pancreatic juice cytology was assessed in combination with the profiling of miRNA biomarkers [131]. In addition, a second study on pancreatic juice has recently shown that a panel of miRNAs (miR-21, -25, -16) in combination with serum CA19-9 level can improve the sensitivity and specificity in differentiating between PDAC patients and healthy control (75.5% and 86.7 respectively), when compared to serum miRNAs plus CA19-9 [27].

Other miRNAs have also been discovered as biomarkers to predict elevated risk for metastasis. The expression of 6 miRNAs (miR-155–5p, -196b-5p, -365a-5p, -629–5p, -675–3p and -92b-3p) in tumour biopsies from human PDAC patients were found to be significantly correlated to higher risk of lymph node metastasis, and improved the accuracy in diagnosing lymph node metastasis in combination with serum CA19-9 levels [136]. Another study indicated that serum miR-4669 can differentiate the presence of lymph node metastasis from healthy control and PDAC patients that have absence of lymph node metastasis, something which cannot be determined with CA19-9 [99]. As such, utilising miRNA biomarkers present in patient samples may be able to accurately provide more information about disease progression without the need for invasive procedures.

### Prognostic value

The initial evidence supporting the potential of miRNA’s prognostic value was Takamizawa et al*.* who found consistently reduced *let-7* expression levels in lung cancer patients significantly associated with worse survival outcomes post-tumour resection [147, 148]. Following this, a large clinical study involving a total of 686 patients, showed that high miR-21 expression in PDAC tumours was significantly correlated with shorter overall survival, along with other clinical features of advanced development, such as high tumour grade and presence of lymph node metastasis [149]. However, those patients with high miR-21 expression are also predicted to have an increased survival benefit after receiving gemcitabine-based adjuvant chemotherapy as compared to low miR-21 patients. With extensive evidence that correlates miR-21 overexpression with chemotherapy resistance [150, 151], this indicates that miRNA expression might be able to assist in guiding clinical treatment regimens for different patients.

Increasing amounts of evidence suggest that several miRNAs are also altered after surgical resection, and/or chemotherapy, and are typically associated with risk of recurrence. In a cohort of 26 PDAC patients who underwent pancreaticoduodenectomy, serum miR-99a-5p and miR-125b-5p were upregulated following surgery and significantly associated with shorter progression-free survival [152]. A risk score developed incorporating expression of miR-181b/d and miR-575 was validated for use in assessing the risk of locoregional recurrence and worse overall survival in PDAC patients receiving different treatments [153].

Changes in circulating miRNA expression are also useful as a predictor or indicator of treatment response. For example, serum miR-373-3p and miR-194-5p were found to be overexpressed after one cycle of FOLFIRINOX treatment in PDAC patients whose disease progressed compared to patients with stable disease [154]. Further validation of these miRNA biomarkers in PDAC patients are currently in progress with 9 recruiting clinical trials registered on ClinicalTrials.gov worldwide (Table 5). Two recently completed clinical trials that aimed at studying the diagnostic value of miRNA have yet to publish the trial outcomes.

**Table 5:** Currently recruiting/completed PDAC clinical trials using miRNA as a diagnostic and/or prognostic biomarker

Clinical trial ID	Study status	Study objective	Location	Result/expected completion year
NCT05556603	Recruiting	To investigate the sensitivity and specificity of blood miRNA detection for the detection of PDAC	China	2029
NCT03311776	Recruiting	To identify potential diagnostic, prognostic and predictive biomarkers by measuring a range of circulating molecules including miRNAs	Denmark	2035
NCT03886571	Recruiting	To investigate cell-free and exosomal miRNA as biomarkers in tissue and plasma from healthy controls and patients with PDAC, pancreatic neoplasms, pancreatitis and diabetes	United States	2024
NCT04158635	Recruiting	To assess safety profile of Bosentan with profiling circulating miRNA to assess dose response and identify potential biomarkers	United States	2026
NCT04406831	Recruiting	To investigate the potential of circulating miRNA to allow early diagnosis and predict response to treatment	United States	2027
NCT05275075	Recruiting	To assess the changes in miRNA and mRNA expressions in tumours from PDAC patients who received surgical resection and their association with cachexia	United States	2028
NCT05495685	Recruiting	To investigate the sensitivity and specificity of blood miRNA detection for the detection of PDAC	China	2024
NCT05633342	Recruiting	To develop a multi-cancer screening test through analysing potential blood biomarkers including miRNA and other cell-free nucleic acids	Singapore	2025
NCT06139042	Recruiting	To assess the use of a combination of assays measuring cell-free DNA methylation, serum protein and miRNA as a diagnostic biomarker for early liver, biliary tract, and pancreatic cancer detection	China	2025
NCT02807896	Completed	To investigate the effectiveness of a diagnostic chip with integration of miRNA and other biomarker analysis in pancreatic and bile duct cancer	Korea	Completed in 2016, result unavailable
NCT02504333	Completed	To investigate miRNA expression levels and their correlation with treatment response and other blood biomarkers in PDAC patients receiving Nab-paclitaxel (Abraxane) and Gemcitabine followed by modified folinic acid, fluorouracil, oxaliplatin (FOLFOX)	Spain	Completed in 2021, result unavailable

An observational study in China is estimated to involve over 7,000 patients including newly diagnosed PDAC patients, patients with non-malignant pancreatic disorders, participants with high risk of PDAC and healthy control individuals in order to identify dysregulated circulating miRNAs that may indicate early onset of PDAC as well as other malignancies (NCT05556603). Similar studies to identify diagnostic, prognostic and predictive miRNA biomarkers are underway in Denmark (NCT03311776) and the United States (NCT03886571).

Interestingly, a phase I clinical trial that aims to elucidate the safety of using an endothelin antagonist Bosentan with PDAC standard-of-care gemcitabine and nab-paclitaxel, is profiling circulating miRNAs to assess treatment response and analysing miRNA expression in tissues to identify potential prognostic biomarkers (NCT04158635).

There are several limitations that will need to be overcome before miRNA biomarkers can be widely implemented in the clinic. The currently published data on miRNA expression, both in pre-clinical models and patient specimens, are often inconsistent and sometimes contradictory. For instance, miR-10b is found to be downregulated in PDAC cell lines and tumours [43] but the opposite was found in other studies utilising human PDAC tumour and pancreatic lesion samples [155, 156]. Different miRNA expression profiles can be found even in the same patient cohort, possibly due to the heterogeneity of the disease in question. This creates an obstacle in using miRNA as a reliable diagnostic tool, which has resulted in more recent studies developing panels of miRNA signatures that are used in combination with serum CA19-9, hoping to increase the sensitivity and specificity in PDAC detection. Some of these discrepancies could be due to using different miRNA extraction protocols. Emerging studies have re-emphasised the need to standardise protocols to achieve consistent and comparable results, and novel protocols are being developed to achieve better EV isolation [157, 158]. Considering the limited availability of patient specimens, efforts have been made to increase miRNA detection and characterisation from low volumes, with some approaches now requiring as little as 20 µL of plasma/sera. While there is currently an increased interest into miRNAs in a broader range of bodily fluids, it is important to note that miRNA expression typically varies between sample type. For example, there are large differences between tissues and plasma [81], therefore we should be cautious on referencing previous sequencing results from different samples of origin. Another major controversy on the use of miRNA as diagnostic marker is that miRNA in the circulation may not tumour specific and could be altered due to malignancies originating in other organs. A good example is oncogenic miR-21 which is found to be overexpressed in many other cancers other than PDAC, such as lung and colorectal cancer [159]. Future studies in developing miRNA diagnostic biomarkers will need to focus on improving the specificity of its detection in PDAC before miRNA can be routinely used in the clinic.

## miRNAs as therapeutic targets in pancreatic cancer

In recent years, an increasing number of dysregulated miRNAs have been discovered in cancer and extensive evidence suggested that they directly regulate cancer-associated mRNAs and downstream signalling pathways. Targeting cancer through manipulating miRNAs is appealing since it might allow personalised treatment based on individual’s specific gene or miRNA expression profile and could potentially overcome chemotherapy resistance especially in PDAC. There are generally two ways in targeting miRNAs: miRNA inhibition and miRNA replacement/mimicking [160]. Inhibiting miRNA can be achieved using small molecules such as anti-miRNA oligonucleotides (AMOs), antisense oligonucleotides (ASOs) and miRNA sponges that bind to target miRNA via a complementary base sequence and prevent them from binding to target mRNA. On the other hand, miRNA replacement therapy involves the use of synthetic miRNA or miRNA mimics to inhibit tumour-promoting mRNA activities by post-translational suppression. For example, miR-506 has previously been established as a tumour suppressing miRNA in PDAC, and researchers have shown that by transfecting a tumour-suppressing miR-506 mimic into cancer cells in vitro or by delivering nanoparticle packaged miR-506 mimic into a mouse xenograft model, that it led to an anti-proliferative and apoptotic effect in cancer cells, thereby suppressing tumour growth in vivo [161]. More excitingly, multiple tumour suppressing effects of miR-506 have been uncovered ranging from altered cell cycle progression, promotion of senescence, increased autophagy and reactive oxygen species (ROS) generation, due to miR-506’s ability to bind and regulate several oncogenic mRNAs, including STAT3 [162], EZH2 [163], CDK4/6 [164] that are all part of important downstream pathways mediating cell cycle, survival, and apoptosis. This work illustrates how powerful miRNA can be as a therapeutic strategy to target multiple cellular processes at once.

MicroRNAs can also be modified to improve the efficacy of current standard-of-care chemotherapy. When miR-15a was integrated with 5-fluorouracil (5-FU-miR-15a) and used to treat pancreatic cancer cells, not only did it significantly inhibit cancer cell proliferation, but it also sensitised the cancer cells to gemcitabine [44]. More importantly, treating PDAC metastatic mice with 5-FU-miR-15a alone significantly reduced metastatic growth in vivo compared to gemcitabine monotherapy, and an even stronger response was observed in the 5-FU-miR-15a plus gemcitabine combination treatment group. In a separate study, a combination therapy of nanoparticle CXCR4, siKRAS^G12D^ and anti-miR-210 inhibited cancer cell interactions with pancreatic stellate cells to slow tumour progression and reduce stromal desmoplasia to increase drug delivery in pre-clinical mouse models of PDAC [109].

Despite promising results in pre-clinical models, effective delivery of miRNA-based therapies to the tumour site without systemic toxicity remains a major concern in bringing miRNA therapeutics to the clinic. Free miRNAs rapidly degrade and cannot be taken up by cells, so miRNAs must be packaged into and transported in vehicles such as non-viral carriers (liposome, nanoparticle, EV, minicell), viral vectors (retroviral, lentiviral) or chemically modified to be conjugated with other molecules. To date, only two miRNA-based therapeutics for cancers have been tested in clinical trials and both have utilised different vehicles for delivery. In 2017, the first clinical trial (NCT02369198) testing a novel technology named TargomiRs commenced. TargomiRs are bacterial-derived minicells that contain miRNA mimics and are designed to recognise a specific target. In this study, the TargomiR MesomiR-1 contains tumour suppressive miR-16 and antibodies that recognise epidermal growth factor receptor (EGFR), which is often found overexpressed and associated with worse prognosis in non-small cell lung cancer (NSCLC). Restoring levels of miR-16 was previously found to ameliorate tumour progression in vivo. Early results showed that intravenously injected MesomiR-1 was safe in patients with no adverse effects, and this study is now moving forward to a phase II trial [165]. Unfortunately, the second miRNA therapy that was tested for safety, the liposomal miR-34a mimic (MRX34) caused severe immune-related reactions and the study was suspended prematurely [166]. This was thought to be as a result of the miRNA activating the innate immune system through stimulating inflammatory cytokine production and the immune system against the liposomal vehicle [167, 168].

Given that the survival rate of PDAC has not significantly improved over the past decades, the potential for therapeutic miRNA-based approaches targeting multiple cancer-associated mRNAs is particularly appealing to treat this multifaceted disease. However, this may also represent a double-end sword as miRNA-based therapies could illicit broad off-target effects leading to adverse life-threatening outcomes. Future studies will be required to focus on the mode of delivery that ensures target specificity and low toxicity before miRNA therapy can be routinely used in the clinic.

## Future directions and challenges in miRNA research in pancreatic cancer

As the field of miRNA research expands, there have been many exciting findings regarding miRNAs playing important roles in cancer. Advancements in technologies have also accelerated our current understanding of this class of small RNAs. As mRNAs are the direct target of miRNAs, it is ideal to co-profile both miRNAs and mRNAs in the same sample to begin correlating change in expressions of both and the inter-dependency of the two. While traditional bulk RNA sequencing is limited and ignores crucial elements such as tumour heterogeneity, the advent and increased availability of single cell sequencing is likely to become more widely incorporated into the study of both miRNA and mRNA transcriptomes specific to individual cell types [169]. This will likely be further improved by platforms such as CSmiR which helps to identify target mRNAs of cell-specific miRNAs, and incorporates cell-to-cell communication based on the miRNA-mRNA interactions [170]. Some sequencing data from patient samples, such as TCGA which have been made publicly available online accessible via the Xenabrowser [171], present a rich source of miRNA data and have been utilised by many to study differentially expressed miRNAs in cancer. Since miRNAs often have more than one target, and multiple miRNAs are able to target the same mRNA, other online resources continue to be developed to assist in predicting potential mRNA targets for further investigation (Table 6).

**Table 6:** Online resources available for miRNA research

Databases	Functions	References
miRBase	Summarises all the published miRNA sequences and annotations	[3]
TargetScan	Predicts miRNA targets	[31]
miRWalk	Predicts miRNA targets based on a machine-learning algorithm, TarPmiR Allows searches on the interactions between genes in signalling pathways or biological processes in diseases	[172]
miRDB	Predicts miRNA targets by a bioinformatic tool, MirTarget	[173]
miRTarBase	Stores published studies that functionally validated miRNA targets	[174]
miRCancerdb	Stores information on miRNA to gene/protein expression association in cancer, based on TCGA and TargetScan data	[175]
MiREDiBASE	Provides information on miRNA editing and modification that may affect miRNA biogenesis	[176]

While sequencing data are able to reveal a broad overview of miRNA and RNA changes in samples, it requires functional validation to confirm biological effects. Therefore, miRTarBase is particularly valuable since it records all miRNAs and targets validated in functional assays such as luciferase reporter assays, western blot and quantitative polymerase chain reaction (qPCR) [174]. Due to the intrinsic cellular heterogeneity of many solid tumours and to overcome the limitation of bulk tissue sequencing, spatial detection of miRNA expressions has recently received a lot of attention to being to investigate and map where a miRNA of interest is localised within a specific tissue. Traditionally this has be achieved via fluorescent in situ hybridisation on formalin fixed paraffin embedded (FFPE) samples [177]. However recent advancements in miRNAscope technology now allows co-visualisation of miRNA, RNA and proteins simultaneously [178]. In fact, integrating miRNA analysis with other -omics data is becoming more widespread and is allowing researches to gain a more holistic all-round understanding of diseases [179–183].

## Conclusion

Despite remarkable progress in research which has allowed better understanding of PDAC, the survival rate has shown little improvement in the last three decades. This is mainly due to late diagnosis and resistance to therapy. Increasing evidence has shown that deregulated miRNAs directly regulate cancer-associated mRNAs, and significantly contribute to PDAC initiation, progression, and treatment resistance. Ongoing research has been focused on utilising miRNA as a diagnostic and prognostic biomarker, as well as manipulating miRNA expression as novel therapy. While encouraging results have kickstarted several clinical trials started worldwide on using miRNA as a diagnostic marker, systemic toxicity remains the major hindrance for the translation of miRNA-based therapies into the clinic. Future research should continue to uncover and map the biological significance of deregulated miRNAs in solid tumours, and explore their potential in early diagnosis, and prognosis as well as developing novel approaches by which they can be targeted a treatment modality to improve patient outcome and survival.
